# Professional Hobbyism

**Published:** 1878-05

**Authors:** Henry E. Beach

**Affiliations:** Ex-President of the Tennessee State Dental Association.


### ARTICLE V.

### *Professional Hobbyism.*

BY HENRY E. BEACH, D. D. S.,

Ex-President of the Tennessee State Dental Association.

Mr. President, and Gentlemen of the Virginia State Dental Association:

It was my intention to have been with you at your meeting in Richmond, but as I find that circumstances will make it impossible, I send you instead, a paper, which I trust you may not find uninteresting. I select for my theme—

### PROFESSIONAL HOBBYISM.

It is a fact too commonly observed among all professions that extremists arise, who pursue their hobbies with one idea so constantly before them as to shut out from their vision all else. They shoot off into some pet scheme of their own, or if not sufficiently original, into the scheme of some one else's, crying "Eureka; I have found the highway to perfection." We see such often, as we see meteors darting along the horizon for an instant, shining, but suddenly disappearing, leaving only a light streak to denote their late track. Like the meteor he is gone forever, unless he as suddenly discovers another "Pearl of great price."

To leave metaphor and be more practical: The advocates of gold as the only fit material for filling teeth, plant themselves on this precious metal, and shout aloud on every occasion, whether appropriate or not; "Away with tin, away with amalgam, I can fill any tooth with gold that can be filled at all." Any material other than the yellow metal is as revolting to their sensibilities as cannibalism, and should not be tolerated for a single instant. They thus make a hobby of the most desirable of all materials for filling teeth. While common sense and the observation which nature furnished them,—but which they have never rightly used—should have taught them that these less costly and in many cases, less desirable materials, have their place in the catalogue of useful and desirable fillings, and without them many thousands of teeth that are now doing good service would be monuments of the skill of the cheap dentist who makes "extracting" and "rubber" a specialty, and who sees no help for a tooth which "has a hole in it."

Sub-dividing this golden subject, arises the *cohesive* dentist, who describes his methods in long articles, giving all the minutest details, from the preparation of the cavity to the final polishing touch, which description would be excellent, did it not begin, continue and end in the arrogant assertion that "if others will learn of *me*, follow *my* advice in detail, regardless of any contra-indications which may be present, there will be no more failures, no rat-holes, no checked enamel from heavy malleting, no giving away of cervical walls of proximal cavities, but all will glide smoothly on, and the work endure to the end of time, as a monument of the skill and good sense of the distinguished operator."

Another sub-division of the golden field is worked by the exclusively *non-conhesive* advocate; he who uses nothing but Abbey's Soft Foil No. 4, and never makes a failure. He has a peculiar way, all his own, of tucking it under and working it in, so as to combine all the good there is in gold in any method. *He* would not suffer any other than non-cohesive gold to come near his office, and with all the egotism of a hobby-rider he cries aloud: "come unto me all ye dentists and learn wisdom that will give you success through life, and leave you a crown of laurels when you pass away."

Tin has its advocates. So has Amalgam, Oxy-chloride of Zinc, Wood's Metal, Gutta-percha, &c. Mechanical Dentistry too is by no means free from its hobbies. But I forbear to detail. All these egotists and exclusivists seem to forget or to ignore the great principle that underlies success in any avocation in life: that there must be a proper adaptation of means to ends. We should never forget that "intelligent diagnosis must precede manipulation."

It may seem a bold assertion to make, but it is nevertheless a self-evident fact, that he who has a stereotyped rule by which he is governed, regardless of idiosyncrasies, temperament, &c., is a quack in the broadest sense of the word, and can no more fill the requirements of the the dental profession, than a butcher can perform an operation in staphyloraphy. Then let us unshackle our minds, divest ourselves of every hobby, and look well to our diagnosis, and carefully make up our minds as to the prognosis, before we venture even to suggest what is best to be done in the premises.

To do this, many things are to be considered. We must consult the general health, idiosyncrasies, if any, the age and temperament, the character of the secretions, and occult sources of disease calculated to effect them ; habits of cleanliness, the character of the operation and of the organs to be operated upon: on each one of which subjects I would like to speak of separately, if time would permit, but I can not. When we have done this why not divest ourselves of all pet theories, and take a conservative view of the situation, then if the case is one demanding a gold filling, is a gingival or crown cavity, with strong walls, fill with non-cohesive cylinders ; or if a compound proximal cavity in the molars or bicuspids, use cylinders at the cervix or until nearly full and finish with cohesive gold, and if a compound complicated cavity in any tooth, fill with cohesive alone, retaining with screws if necessary, and thus get the best results by an intelligent adaptation of the different preparations of gold, all of which are good in their place. If the case, owing to the class of teeth, demands tin, hesitate not to use it with much confidence, for in many instances, it surpasses anything known as a filling. Many teeth can be saved with it, when gold would positively hasten their destruction. Amalgam though much abused has its place too. In badly decayed molars, and out of the way places, where you are unable to use anything else durable, or your patient is not able to pay for large contour gold fillings, nothing is so good as amalgam. As it is in operative dentistry so it is in mechanical, but gentlemen, we need all the different appliances in mechanical dentistry known to us, and should discard none of them. Gold, platina, rubber and celluloid all come in at their proper place, and our duty is to know where that place is. The intelligent farmer would laugh at the idea of preparing his soil for different crops in the same way, or planting all kinds of seed on the same quality of soil, yet there are men in our profession who are doing practically this very thing, and the sooner we stop such a practice, the better it will be for us and those who need our services. Then let us be careful that we nurture no pet theory to the exclusion of truth and light but by a rational system of ecclecticism exclude, combine and discriminate, and thus accomplish the end of our mission, the preservation of the teeth of our patients. I believe there are men now practising dentistry who would rather lose the teeth of their patients through gold, than save them by the use of amalgam, just as there are men who have tried to work for years after their sight began to fail without glasses, fearing to confess that they could not see, though betraying their defective vision at every turn. They see their failures, but professional hobbyism will not let them confess their errors, until in time the truth itself seems error, and error assumes the guise of truth.

